# FM-Net: A New Method for Detecting Smoke and Flames

**DOI:** 10.3390/s25175597

**Published:** 2025-09-08

**Authors:** Jingwu Wang, Yuan Yao, Yinuo Huo, Jinfu Guan

**Affiliations:** 1 School of Electronic and Information Engineering, Anhui Jianzhu University, Hefei 230601, China; wangjingwu@tsinghua-hf.edu.cn (J.W.); yaoyuan@stu.ahjzu.edu.cn (Y.Y.); 2 Hefei Institute for Public Safety Research, Tsinghua University, Hefei 230601, China

**Keywords:** smoke and flame detection, feature pyramid network, deep learning

## Abstract

Aiming at the core problem of high false and missed alarm rate and insufficient interference resistance of existing smoke and fire detection algorithms in complex scenes, this paper proposes a target detection network based on improved feature pyramid structure. By constructing a Context Guided Convolutional Block instead of the traditional convolutional operation, the detected target and the surrounding environment information are fused with secondary features while reconfiguring the feature dimensions, which effectively solves the problem of edge feature loss in the down-sampling process. The Poly Kernel Inception Block is designed, and a multi-branch parallel network structure is adopted to realize multi-scale feature extraction of the detected target, and the collaborative characterization of the flame profile and smoke diffusion pattern is realized. In order to further enhance the logical location sensing ability of the target, a Manhattan Attention Mechanism Unit is introduced to accurately capture the spatial and temporal correlation characteristics of the flame and smoke by establishing a pixel-level long-range dependency model. Experimental tests are conducted using a self-constructed high-quality smoke and fire image dataset, and the results show that, compared with the existing typical lightweight smoke and fire detection models, the present algorithm has a significant advantage in detection accuracy, and it can satisfy the demand for real-time detection.

## 1. Introduction

Fire poses a major threat to public safety and social development, and its prevention and control system is facing new challenges in the complex environment of modern cities. According to the statistics of the China National Fire and Rescue Administration, from January to October 2024, 774,000 fire accidents were reported, resulting in 1559 deaths, 2202 injuries, and CNY 4.76 billion in direct economic losses. The causes of accidents include the dynamic coupling of multiple factors such as electrical system failures, improper use of fire, and climatic anomalies, and such nonlinear interactions significantly increase the decision uncertainty of traditional point sensors. In the context of the extensive use of new composite materials in modern buildings and the increasing complexity of spatial structures, the fire evolution process presents significant non-stationary characteristics, making it difficult to achieve early and accurate warning with fixed-threshold contact-based detection systems. Therefore, the development of non-contact fire detection technology with real-time response capabilities has become a key research direction to enhance the resilience of urban safety.

The continuous development of computer vision technology has brought new breakthroughs in the field of fire detection, and its non-contact perception characteristics and dynamic feature resolution capability provide a new technical path for early fire warning. In the field of computer vision, the existing research mainly focuses on chromaticity spatial distribution, texture complexity and the motion vector field of flame and smoke to build a multi-dimensional feature discrimination system. The color-associated fuzzy affiliation model proposed by Li H. et al. [1]. establishes the weighted affiliation function of three-channel RGB through the gray scale association theory, which has a high recall rate in small sample scenes but a lack of completeness in color space modeling, only divided into three backgrounds: red, green, and blue. This reflects the essential limitations of traditional image fire detection methods in general, which fail to break through the technical barriers between static color space modeling and dynamic feature spatio-temporal decoupling.

In the field of machine learning-driven visual recognition of fire, the research paradigm mainly revolves around feature engineering and classifier co-optimization. This is usually performed by constructing a hierarchical feature space to achieve linear separability of flame smoke in limited dimensions. Bai et al. [2]. reconstructed the fire data distribution and built a logistic regression model by using over-sampling, under-sampling, hybrid sampling and synthetic methods. The Bayesian network constructed by Liang et al. [3]. introduce conditional probability tables to portray risk factor associations. LU et al. [4]. use multi-task-based learning for fire detection, integrating the advantages of multi-tasking to improve the accuracy of detection. All these cases expose the contradiction between feature engineering and model generalization ability in traditional machine learning methods. When facing the sudden change of viewpoints or complex interference sources in industrial scenes, the discriminative power of manually designed features shows exponential decay, and computer vision monitoring methods based on machine learning do not have strong transferability.

Deep learning algorithms automatically extract deep features from image data by constructing convolutional neural networks, which show advantages over traditional methods in fire feature learning. Its core value lies in the establishment of a nonlinear mapping relation from pixel space to semantic space and the asymptotic completeness of the feature transformation by stacking convolutional kernels. Zhang et al. [5] added DenseNet to the feature extraction network to improve the reuse rate of flame features in an attempt to better monitor real-life fires. Although these detection methods automate fire detection to a certain extent, they are not lightweight enough, and the improved model inference speed decreases from 27.2 FPS in the original YOLOv3 to 26.0 FPS, implying that the computational complexity may not be optimized and may still not satisfy the requirements of low-power scenarios. Chen et al. [6] designed the SPPCSPC (Stage-Placed Spatial Pyramid) architecture, which realizes multi-scale perceptual field fusion on a ResNet-50 backbone by constructing a cross-layer feature interaction channel, and it effectively mitigates the feature offset phenomenon caused by scale change and improves the robustness of smoke target detection, but the number of parameters of the improved model reaches 35.4M, which is far more than the 20.8M of the original YOLOv5m and even higher than YOLOv7 (36.48M) and TPH-YOLOv5 (45.37M). This indicates a significant increase in model complexity.

However, there are still some shortcomings in the existing methods, and their limitations are mainly reflected in three aspects: (1) insufficient multi-task co-optimization ability, poor scene adaptation ability of the existing detection models, and inconsistent sensitivity to smoke and flame, which makes it difficult to achieve early warning; (2) lack of sensitivity to small targets and exponential degradation of detection accuracy when the percentage of flame and smoke area decreases; and (3) multi-scale adaptation defects. The current algorithms have difficulty in adapting to such scale changes, as flame and smoke are usually attached to objects with huge differences in scales.

To address the above problems, this study proposes a dynamic fire detection framework based on multi-task learning, and the main contributions of this paper are as follows:

(1) Enhancing the generalization ability of the model by constructing a dedicated dataset covering flame, smoke and complex backgrounds and adopting an adversarial filtering enhancement strategy.
(2) Introducing a Context Guided Convolutional Block to achieve structured decoupling of the feature space and progressive dimensionality simplification; optimizing the performance of capturing the details of the detected target by combining this with a Poly Kernel Inception Block; and solving the problem of dynamic characterization of the smoke diffusion by using the Manhattan Attention Mechanism Unit to model the long-distance dependency relationship between pixels.
(3) A lightweight network architecture is constructed to achieve significant improvement in detection accuracy and real-time performance, which provides reliable technical support for early fire warning in complex scenarios.

## 2. Related Work

### 2.1. Fire Detection

Non-contact fire detection technology with real-time response capabilities, as a key technical support system for the construction of smart and safe cities, is centered on the accurate capture of early smoke and flame characteristics.

The current deep learning-based fire detection algorithms mainly present two research paradigms: the two-stage detection framework [7,8,9] represented by Faster R-CNN and the single-stage end-to-end detection architecture represented by the YOLO series [10,11,12,13,14]. Theoretical analysis and experimental studies show that although the two-stage algorithm has advantages in target localization accuracy, its cascading processing process leads to higher computational complexity, which severely restricts the real-time response performance. In contrast, the single-stage algorithm achieves the synergistic optimization of detection accuracy and inference speed by introducing a global context-aware mechanism and a multi-scale feature fusion strategy. This feature makes the single-stage algorithm show significant engineering value in scenarios that need to satisfy strict real-time constraints, such as smart city video surveillance networks, especially when dealing with complex conditions such as dynamic fire source spreading and multi-target staggered motion. The parallelized feature extraction capability of the single-stage algorithm can greatly improve the throughput of the detection system. For example, Talaat et al. [15] applied the YOLO algorithm to smart city fire detection, using a hierarchical structure to transmit fire features layer by layer to construct a smart fire detection system (SFDS). Huang et al. [16] proposed a lightweight forest fire detection method based on the YOLOX and defogging algorithms. The method uses a dark channel before image defogging to obtain fog-free images and is improved by decreasing its weight for forest fire detection in fog-free images. Zhang et al. [17] used a Context-Oriented Multi-Scale Neural Network for fire image segmentation. This network employs convolutional operations to leverage the relationships between all pixels in the feature map, thereby expanding the network’s receptive field and enabling it to more effectively distinguish between fire and non-fire pixels. Wang et al. [18] added a CA attention mechanism before the SPPF layer in the YOLOv5s backbone network to enhance the model’s attention to spatial and channel information features, thereby improving the spatial relationships of matched features. This ultimately reduced the number of model parameters by 2.46 million and improved detection accuracy by 3.1%.

To improve the robustness of smoke and flame detection in complex environments, such as extreme lighting variations, severe occlusion and background interference, researchers are actively introducing the Transformer model. Compared to CNNs, which mainly rely on local receptive fields, Transformer’s self-attentive mechanism can directly model long-range dependencies between arbitrary pixels in an image, which is crucial for smoke and flame detection. Smoke has diffuse, semi-transparent and fuzzy boundaries, and its morphology is highly dependent on long-range pixel associations, while flames are susceptible to partial occlusion or confusion with complex backgrounds, such as evening sunsets and lights, and require global information for discrimination. To address the semi-transparency and fuzzy boundary problem of smoke, Wang et al. [19] proposed the frequency domain Transformer branch, which extracts the low-frequency semantic structure by Fourier transform and is complemented by a multilevel high-frequency perception module to capture the edge details. For efficient fusion of multilevel features, Cao et al. [20], on the other hand, constructed a parallel architecture of Transformer and U-Net and designed an innovative BiFusion module. However, the self-attention mechanism at the core of Transformer suffers from O(n^2^) computational complexity, which becomes a significant performance bottleneck when dealing with long sequential inputs.

These empirical studies show that the single-stage detection framework has initially constructed a fire detection paradigm to meet the real-time security needs of smart cities through algorithm–hardware co-optimization, but its robustness under extreme lighting conditions and complex occlusion scenarios still needs to be improved by a self-supervised learning mechanism.

### 2.2. Attention Mechanisms and Manhattan Distance

Traditional convolutional neural networks extract features through local receptive fields, but when facing complex scenes, local features are often insufficient to capture global contextual information. To compensate for this deficiency, the attention mechanism was created to enhance the expressive ability of the model by assigning different weights to features at different locations.

Attention mechanisms are used to improve feature selection and information aggregation in target detection tasks. Common attention mechanisms include channel attention [21], spatial attention [22], and self-attention [23]. Channel attention mechanisms enhance the sensitivity of the network to important features by assigning different weights to each channel. Spatial attention mechanisms further enhance the ability to model local information in an image by assigning different weights to different regions in the spatial dimension.

In addition, improving model performance by introducing Manhattan distance [24] is also an important research direction in the field of target detection. By introducing the Manhattan distance (L1 distance) instead of the traditional dot product similarity computation, which aims to capture the difference features between sequence elements, the trained model can focus on the key regions in the image and suppress irrelevant information, thus improving the accuracy of detection, and the method is especially suitable for detecting spatially or locally dependent bodies such as flame and smoke.

In smoke and fire detection, the boundaries of smoke are often blurred, the contours of flames change with the morphology of combustible materials, and the morphological features presented vary in different scenarios; thus, how to accurately capture the salient regions of smoke and flames has become the key to improve detection performance. To this end, this paper provides a new similarity metric perspective for the early fire detection task by incorporating the Manhattan distance, which shows potential in structured and spatial data, albeit with a trade-off in computational efficiency.

## 3. Methodology

**Figure 1:**
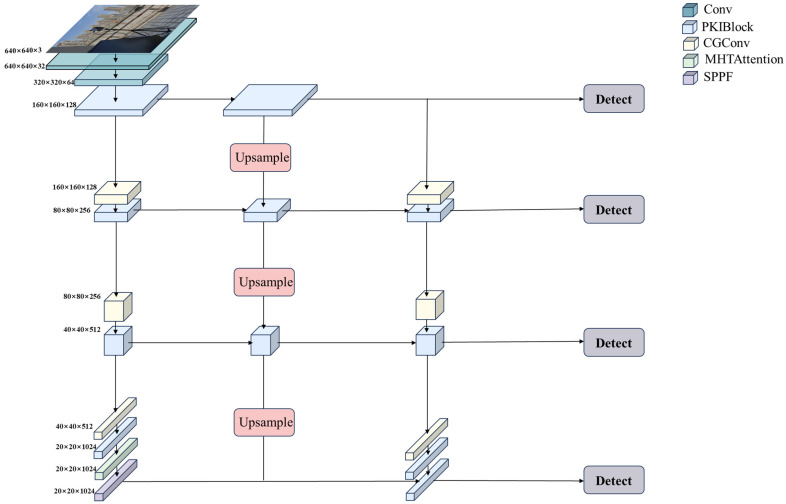
The overall architecture of the network model.

The deep learning network model designed in this paper uses a feature pyramid structure as the backbone network for target detection. Considering the inconsistency in the size of the firework images in the dataset, it is particularly important to be able to extract features at multiple scales when processing such images. The feature pyramid captures both detailed and global information in an image by constructing a series of layers, each representing features at a different scale. As the feature pyramid changes layer by layer, the sensory field representation of the feature maps has different characteristics, as well as differences in the level of abstraction of the information it conveys. Shallow feature maps are usually more suitable for detecting small targets due to their smaller receptive fields and limited effective information, while deeper feature maps with larger receptive fields are more suitable for large target detection. In the model, we retain the shallow features while sending them as input data to the deeper feature layer and fuse them with the deeper feature map for feature fusion. This approach allows for deeper semantic information to be combined with lower-level detail information, which helps to solve the poor feature representation capability of the lower-level feature map and the loss of information in the deeper feature map. The overall architecture of the model is shown in Figure 1.

### 3.1. Context Guided Convolutional Block

The central contradiction faced by lightweight networks in smoke and fire detection tasks is between the limited model capacity and computational resources and the high demand for rich contextual information. To address this contradiction, the target detection task needs to classify each pixel, which is highly dependent on understanding the environment around the pixel. For example, determining whether a pixel is “sky” or “ocean” depends heavily on what pixels are above and below it. The core aim of this module is to establish a guided information flow where semantically rich global contextual information can actively and selectively direct, enhance or suppress local detailed features through dynamic attentional modulation. The goal is to endow the lightweight network with powerful scene understanding capabilities so that the learning process of local features is always guided by the global semantics, thus achieving more accurate pixel-level semantic understanding with limited model capacity.

Influenced by the design concept of CGNet [25], this article separately designs a special convolution module. In this paper, assuming that given an image X, local feature extraction is first performed using a convolution kernel of 1 × 1 regular convolution, an operation that accurately captures the thin edge texture of the smoke, the highlighted regions of the flame core, and the color gradient changes at the fire–smoke junction through intensive pixel-level computation, which provide the basis of the subsequent contextual understanding of these local features. The formulaic expression is

(1) $$X_{conv}={Conv}_{1*1}\left(X\right)$$

(2) $$Split\left(X_{conv}\right)=X_{conv_{1}}+X_{conv_{2}}$$

Subsequently, the surrounding context features are extracted using the expansion convolution, which is able to expand the receptive field without increasing the number of parameters to capture a larger range of contextual information, which not only enables the model to perceive the macroscopic morphology of smoke diffusion and the dynamic propagation trajectory of flames under the action of wind but also effectively correlates the detected target with the surrounding environmental context, significantly enhancing the model’s global structure understanding ability in complex scenes. Finally, two feature maps are obtained, and this step is formulaically expressed as

(3) $$X_{conv_{1\_1}}={Conv}_{3*3}\left(X_{conv_{1}}\right)$$

(4) $$X_{conv_{2\_1}}={Conv}_{3*3}\left(X_{conv_{2}}|\;dilation\;rate=2\right)$$

Local and surrounding features are combined through a simple splicing layer and then stabilized by a batch normalization layer to stabilize feature distribution fluctuations due to the low contrast of the smoke image, and an ReLU activation function is used to enhance the nonlinear expression of the flame luminance variations, resulting in joint feature representation with spatial consistency. The formulaic expression is

(5) $$X_{conv_{3}}=ReLU\left(BatchNorm\left(X_{conv_{1_{1}}}+X_{conv_{2_{1}}}\right)\right)$$

Finally, the features are extracted by a global pooling layer, the two fully connected layers are post-connected to generate a weight vector, and the generated weight vector is used to guide the fusion of joint features. This mechanism adaptively enhances the feature response in smoke-dense and flame-flickering regions, while suppressing interfering signals such as water vapor, clouds, or metal reflections to optimize the model detection performance.

Compared to standard convolution operations, its main advantage is that it can more effectively utilize the contextual information of the image and improve the robustness of the model to target scale and position, especially when dealing with complex backgrounds, occlusions, or multi-scale objects.

**Figure 2:**
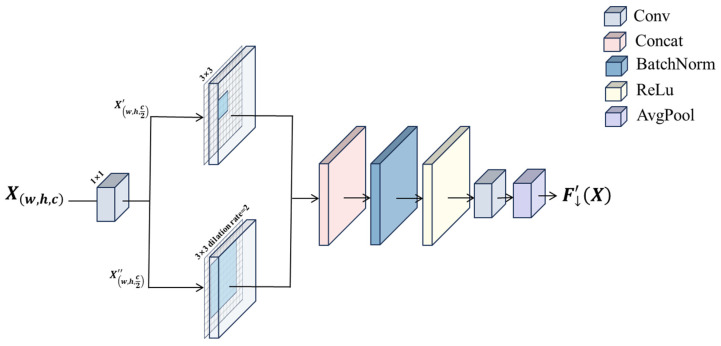
Context Guided Convolutional Block.

The Context Guided Convolutional Block is shown in Figure 2.

### 3.2. Poly Kernel Inception Block

To address the core challenges in target detection in pyrotechnic images, this research focuses on the following key difficulties:

Dynamic changes in the scale of the target. A flame is a high-temperature plasma fluid; its shape, with characteristics dependent on the enflamed material, fluctuates dramatically (such as solid surface spreading and liquid surface ascension, which have very different scale characteristics). Smoke diffusion also occurs due to the composition of the material, ambient temperature and humidity, and other factors, resulting in changes in the scale, from multi-flow to thin dispersion and even to a dense aggregation concentration.

The scene background is highly complex. The actual monitoring scene often contains strong interference elements (such as structural shading of urban buildings, texture confusion with jungle vegetation, reflective interference from glass and other smooth objects, etc.); these complex backgrounds and pyrotechnic targets in terms of the color, texture, and motion characteristics result in a high degree of similarity, leading to positioning drift and misidentification in traditional algorithms.

Therefore, in this paper, we design an aggregated multi-scale perception module, which is embedded into the target detection network through a feature alignment mechanism. The module adopts a two-path parallel architecture: after the input features are split in the channel dimension, the first path extracts local multi-scale features through the Inception [26] Module Plus Block, which integrates multi-branch convolution and a feature reorganization mechanism; the second path captures global semantic information through the Contextual Anchor Attention Block, which utilizes spatial correlation to establish long-range dependency.

The single-path Inception network relies on the width of parallel convolution to extend feature diversity, but it lacks deep interactions between features. The dual-path parallel architecture enables efficient feature reuse and feature exploration compared to the single-path Inception module, which retains historical features and introduces new features, significantly improving feature richness and gradient mobility.

The outputs of the two paths are adaptively fused to form a unified feature representation.

Inception Module Plus Block: This is led by a small convolutional kernel of size 3 × 3 for acquiring local information, followed by a set of larger convolutional kernels of varying sizes interacting in parallel for acquiring multi-scale contextual information, formulaically expressed as

(6) $$X_{inception_{1}}=\sum_{i=1}^{4}{DWConv}_{\left(i*i\right)}\left[{Conv}_{\left(3*3\right)}\left[X_{\left(w,h,\frac{c}{2}\right)}^{\prime }\right]\right]$$

Feature maps are extracted using 3 × 3 convolutions to generate the next layer of feature maps, followed by four depth-separable convolutions to further extract contextual features. Subsequently, the four different feature maps obtained through parallel computation using convolutional kernels of varying sizes are merged to produce the feature map. Finally, a 1 × 1 convolution kernel is used to fuse feature maps at different perceptual scales, enabling the feature map to effectively capture rich global contextual information without sacrificing local detail features, thereby obtaining the final feature map, whose mathematical expression is

(7) $$X_{inception}={Conv}_{\left(1*1\right)}\left[X_{inception_{1}}\right]={Conv}_{\left(1*1\right)}\left[\sum_{i=1}^{4}{DWConv}_{\left(i*i\right)}\left[{Conv}_{\left(3*3\right)}\left[X_{\left(w,h,\frac{c}{2}\right)}^{\prime }\right]\right]\right]$$

The convolution operation of this module achieves deep fusion at the feature level, which broadens the field of view of the features while preserving the delicate texture information.

Contextual Anchor Attention Block: This consists of an average pooling layer, a convolutional layer with a convolutional kernel size of 1 × 1, a depth-separable convolutional layer with a convolutional kernel size of 1 × 2n, a depth-separable convolutional layer with a convolutional kernel size of 2n × 1, a convolutional layer with a size of 3 × 3, and an activation function layer in serial. This module aims to acquire contextual dependencies between distant pixels while enhancing the central features. We take the nth PKIBlock as an example of the following formulaic expression:

(8) $$X_{caa_{1}}={Conv}_{\left(1*1\right)}\left[{Pool}_{avg}\left[X_{\left(w,h,\frac{c}{2}\right)}^{\prime \prime }\right]\right]$$

In this case, we obtain the local region feature map as a 1 × 1 convolution after average pooling, after which we approximate the expression of the large kernel depth convolution using two depth-separable strip convolutions:

(9) $$X_{caa_{2}}={DWConv}_{\left(1*2n\right)}\left[X_{caa_{1}}\right]$$

(10) $$X_{caa_{3}}={DWConv}_{\left(2n*1\right)}\left[X_{caa_{2}}\right]$$

Depth-separable strip convolution is relatively lightweight, and we can achieve a similar effect to 2D depth convolution with a pair of 1D depth convolution kernels with n/2 fewer parameters compared to 2D depth convolution. As the deepening of the model increases the sensory field of the module, we dynamically adjust the convolution kernels of the depth-separable strip convolution layers according to the number of layers n. This design not only enhances the ability of the model to establish long distance pixel relationships, but also the computational cost does not increase significantly due to the strip depth convolution design. The final module generates an attention weight and makes the value of the attention weight lie between 0 and 1 through an activation function. The formulaic representation of the module is as follows:

(11) $$X_{caa}=Sigmoid\left({Conv}_{\left(1*1\right)}\left[{DWConv}_{\left(2n*1\right)}\left[{DWConv}_{\left(1*2n\right)}\left[X_{caa_{1}}\right]\right]\right]\right)$$

Poly Kernel Inception Block: The outputs $X_{inception}$ and $X_{caa}$ of the two sub-modules are subjected to element-by-element multiplication, followed by a network structure similar to residual connectivity, an operation that allows the network to focus more on regions with high weights (detecting the target region), especially when the target is similar to the background, such as in camouflaged target detection, where element-by-element multiplication helps the network to recognize weak differences. In addition, element-by-element addition together with residual concatenation enriches the feature representation of the image by combining different feature information, preserving the feature information from the shallow layer so that the next layer can build on it for further learning. Finally, a 1 × 1 convolution is performed to obtain the final feature map. The complete formulaic representation of the aggregated multi-scale network perception module is

(12) $$F_{\to }^{\prime }\left(X\right)={Conv}_{\left(1*1\right)}\left[\left[X_{inception}⋆X_{caa}\right]+X_{caa}\right]$$

**Figure 3:**
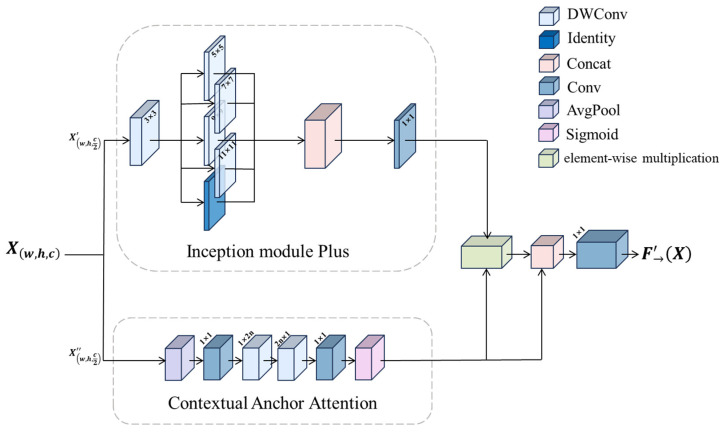
Poly Kernel Inception Block.

The Poly Kernel Inception Block is shown in Figure 3.

### 3.3. Manhattan Attention Mechanism

The Vision Transformer architecture was first proposed to address the training limitations of recurrent models by segmenting images into small, non-overlapping sequences of blocks, and it has achieved great success in many NLP tasks. However, in vision tasks, self-attention, which is a core component of Vision Transformer, lacks a spatial prior and also suffers from high computational complexity due to modeling global information, thus limiting the application of Vision Transformer in the field of computer vision. Researchers have carried out a lot of studies to try to alleviate these problems. In Window Attention, researchers divide the image into non-overlapping local windows and introduce a sliding window mechanism in an attempt to reduce the computational complexity, but the lack of structurally enforced constraints leads to low learning efficiency; in Neighborhood Attention, researchers provide a spatial a priori for every query location based on its content features and dynamically determine a local neighborhood, trying to make the model more spatially adaptable, but they ignore the key contextual information of spatial proximity, so the detection effect is not ideal in some target detection tasks.

Unlike existing methods, the Manhattan Attention Mechanism used in this paper explicitly models spatial dependence in visual tasks by constructing a two-dimensional bidirectional decay matrix. The matrix takes the target pixel as a reference, and the values of its elements monotonically decrease with increasing Manhattan distance, such that the more distant pixels receive more significant attentional weight attenuation. This design achieves a dual advantage: (1) global perception capability: allowing the target pixel to perceive global information and retaining key contextual information of spatial proximity; and (2) distance-aware focusing: dynamically allocating the attention intensity according to the spatial distance and reinforcing locally relevant features to inhibit far-end interference.

To summarize, ordinary attention mechanisms lack explicit spatial constraints, compute globally for all locations, and are susceptible to interference from complex backgrounds, such as clouds, lights, and other flame analogs. A decay matrix is constructed based on the Manhattan distance so that the attention weight of the target Token decreases with increasing distance. This mechanism is consistent with the localized aggregation properties of smoke and flame, meaning that it can focus on the target area more precisely and suppress extraneous background noise.

**Figure 4:**
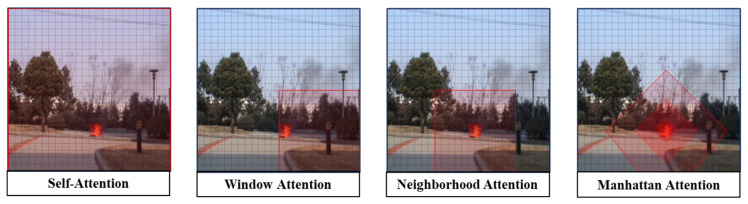
Receptive field of different attentional mechanisms.

Figure 4 demonstrates the spatial prior knowledge obtained by the model as a result of the spatial decay rate of different types of attention mechanisms with the pixel distance.

**Figure 5:**
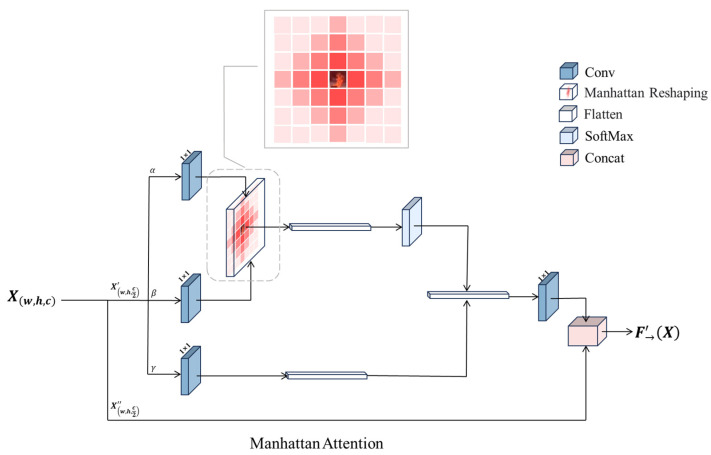
Manhattan Attention Mechanism.

This paper aligns the characteristic parameters of the network model and introduces this module into the network model designed in this paper. The specific model architecture is shown in Figure 5.

The input feature map X is first subjected to a 1 × 1 convolution operation to generate the corresponding α, β, and γ feature representations, respectively. The formulaic expression is

(13) $$Split\left(X_{mhta}\right)=X_{mhta_{1}}+X_{mhta_{2}}$$

(14) $$X_{mhta_{\alpha }}={Conv}_{1*1}\left(X_{mhta_{1}}\right)$$

(15) $$X_{mhta_{\beta }}={Conv}_{1*1}\left(X_{mhta_{1}}\right)$$

(16) $$X_{mhta_{\gamma }}={Conv}_{1*1}\left(X_{mhta_{1}}\right)$$

In the α channel, the input features are downscaled, keeping the spatial dimensions unchanged, thus reducing the computational effort; this step is similar to computing query vectors in traditional attention mechanisms. In the β channel, a similar operation is performed to reduce the dimensionality of the input feature map, and the reduced features are used to compute the spatial dimensionality of the similarity. This step is similar to generating key vectors for similarity computation with query vectors.

Afterwards, in the α and β channels, the generated relative position coding provides information for each position in the pyrotechnic image based on the position of the detected target, which enables the model to adjust the weights according to the relative relationship of different positions when calculating the attention and realizes a kind of spatial a priori perception.

One should generate reduced dimensional input feature representation in the γ channel for feature aggregation after weighting in the attention mechanism; this step is equivalent to computing the value vector in the attention mechanism, which carries the information of the input features and will be multiplied with the weighting matrix to generate the output.

By reshaping the input tensor into a one-dimensional operation, the three features mentioned above are forcefully merged in dimensions other than the number of channels, and then a matrix dot product operation is performed on α and β to get something that corresponds to the class covariance matrix; in the process, the autocorrelation in the features is computed, i.e., we get the relationship of each pixel with all the other pixels in each frame. Then Softmax operation is performed on the autocorrelation features to get the weights from 0 to 1, where the weight function is formally expressed as

(17) $$X_{mhta_{value}}=Softmax\left(\frac{-\left\|X_{mhta_{\alpha }}-X_{mhta_{\beta }}^{T}\right\|}{X_{mhta_{\gamma }}}\right)$$

Finally, we multiply the correlation coefficients, correspondingly, back into the feature matrix γ and then upward expand the number of channels (1 × 1 convolution) and perform the residual operation with the original input feature map X to obtain the output of this module.

## 4. Experiment

### 4.1. Dataset

In this study, some smoke/flame videos were collected from several publicly available datasets, and a large number of experimental videos were collected from ignition experiments carried out in several locations, including real natural scenes and the standard combustion chamber of the Institute. Various fuels such as cotton rope, diesel fuel and beech wood were used in the experiments. During video frame extraction, a strategy of sampling one frame every 4 s was used to ensure significant differences between neighboring samples.

To cope with lighting variations, we captured images of smoke and flame detection at different moments during the day and in different weather conditions. The lighting diversity of the dataset images was increased by artificially introducing lighting variations in the standard combustion room, such as using lights of different intensities, simulating window light, or even switching the lights on and off briefly. To cope with problems such as object occlusion, we labeled occluded flames or smoke even if they were only partially visible, using a bounding box or segmentation mask to cover the visible portion. We explicitly told the model that even if it was partially blocked, that part belongs to the target.

**Figure 6:**
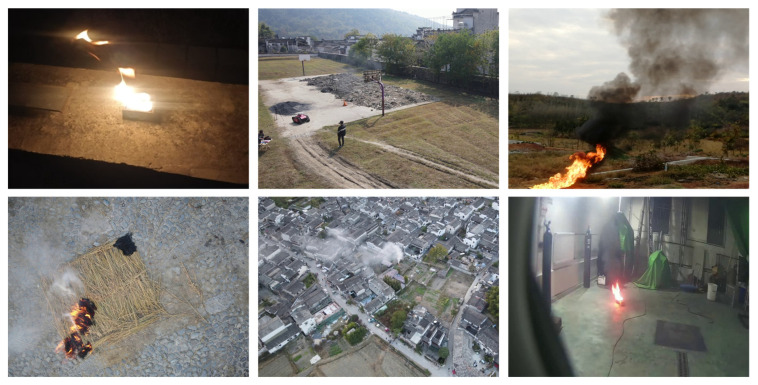
Partial images of the dataset.

A dataset containing 12,625 images was constructed, and some of the samples are shown in Figure 6.

All images were labeled using MakeSense, and the structural composition of the images within the final dataset is shown in Table 1.

### 4.2. Training Environment

The experimental environment for this study is configured as follows: the CPU is Intel i5-13600KF (3.50 GHz), the RAM is 32 GB, and the GPU is NVIDIA GeForce RTX 4070 Ti. The software environment contains CUDA 12.6 and cuDNN. The training strategy uses a dynamic Stochastic Gradient Descent (SGD) optimizer with a cosine decay learning rate, and data enhancement techniques such as mosaic enhancement, image blending and random flipping are applied. The initial learning rate is set to 0.01, the input image size is uniformly adjusted to 640 × 640, and the training batch size is set to 16.

### 4.3. Model Evaluation Indicator

Precision, recall, and mean average precision are commonly used as metrics to evaluate the detection ability of a model.

In this paper, precision is used as a determinant for assessing the accuracy of the model, which is defined as the ratio of the positive predictions (*TP*) of the fireworks to the total number of positive results predicted by the model (*TP* + *FP*). Precision looks at predicted positive cases, as well as true positive and negative cases. When the precision is greater, the smaller the false positives (*FP*), the fewer the number of other categories predicted to be in this category, which can be interpreted as a higher purity of the positive cases predicted. That is, the higher the precision, the fewer the false positives.

(18) $$Precision=\frac{TP}{TP+FP}$$

Recall is defined as the ratio of positive predictions (*TP*) correctly identified as fireworks to the sum of true positives (*TP*) and false negatives (*FN*) predicted by the model. Recall looks at predicted positives and negatives, as well as true positives. As the recall rate gets larger, the false negatives (*FN*) get smaller, meaning that fewer positive cases are predicted as negative cases, which can be interpreted as more positive cases correctly being predicted in their entirety. That is, the higher the recall, the fewer the missed tests.

(19) $$Recall=\frac{TP}{TP+FN}$$

Mean average precision, which is an important indicator of the overall performance of the multi-category target detection model, is obtained by the weighted average of the AP values of all the categories, which can comprehensively reflect the detection ability of the model on multiple categories.

(20) $$AP=\int_{0}^{1}P\left(r\right)dr$$

(21) $$mAP=\frac{1}{n}\sum_{i=1}^{n}{AP}_{i}$$

## 5. Experimental Analysis

### 5.1. Ablation Experiment

**Figure 7:**
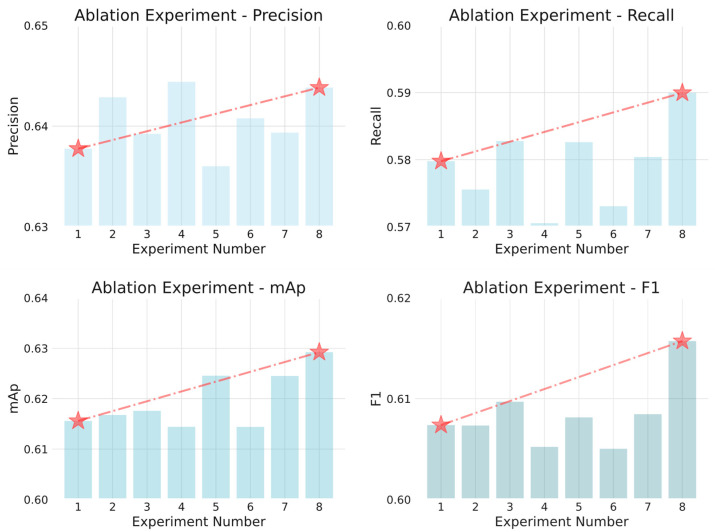
Comparison chart of ablation experiment results.

In order to verify the effectiveness of each structural module proposed in this paper in smoke and flame detection tasks, this study conducts systematic ablation experiments based on the YOLOv8 [27] benchmark model. Three core improvement modules are mainly examined: the Context Guided Convolutional Block (CGConv) for efficient feature extraction and dimensionality approximation, the Poly Kernel Inception Block (PKIBlock) for accomplishing multi-scale target feature extraction and local context capture, and the Manhattan Attention Mechanism (MHTAttention) for performing effective capture of the logical coordinates of target features. The ablation experiments were conducted under conditions where each module was introduced independently as well as jointly, and the performance metrics included precision, recall, class-wide average precision (mAP) and F1 score (F1). In this study, eight ablation experiments were conducted to examine the impact of the various methods. All experimental groups used the same dataset, training setup and training method, and the experimental results are shown in Table 2 and Figure 7.

Ablation Experiment 2 replaces the key convolutional structures in the backbone network with the CGConv module. This module establishes a guided information flow that allows semantically rich global contextual information to be used to extract semantic and edge features of smoke targets at different scales more efficiently through dynamic attention modulation. The experimental results show that the introduction of CGConv improves the mAP to 61.57% and the precision to 64.286%, and the detection accuracy is improved despite the recall decreasing by 0.42%. GFLOPs reduced by 0.7G. This suggests that CGConv has a positive effect on addressing the challenges of smoke- and flame-like targets with varying sizes and blurred shapes in images. Ablation Experiment 3 examines the effect of the PKIBlock module on feature fusion. The module extracts local multi-scale features by fusing multi-branch convolution with a feature reorganization mechanism, which effectively strengthens the feature representation ability of the model in the edge blur region and discontinuous region. Compared with the traditional residual structure, PKIBlock can more accurately detect the morphological features of smoke and flame in complex backgrounds. The experimental results show that the module improves the mAP to 61.759%, recall to 58.277%, and F1 to 60.97%. At the same time, the module reduces GFLOPs by 0.5G, demonstrating its unique advantage in improving edge structure perception capabilities. Ablation Experiment 4 evaluates the smoke/flame detection performance of the MHTAttention module introduced into the backbone network, and the module dynamically allocates the intensity of attention according to the spatial distance, strengthens the locally relevant features to inhibit the distal interference, and thus enhances the detection head’s ability to recognize the low-contrast targets in the complex scene. Under the premise of keeping the overall structure of the model light-weight, MHTAttention will achieve an accuracy of 64.44%.

Ablation Experiment 5, Ablation Experiment 6 and Ablation Experiment 7 test the three modules proposed in Chapter 3 of this paper in two-by-two combinations. The network model shows different degrees of improvement in the four performance indicators: precision, recall, F1 and mAP. Combining the CGConv module with the PKLBlock module improves the recall, mAP and F1 scores, although the precision is slightly reduced. The recall increases from 57.978% to 58.263%, and the increase in the recall obviously pulls the F1 score up, and the mAP also increases to 62.456%. Combining the PKLBlock module with the MHTAttention module resulted in an increase in precision, recall, F1 and mAP, where the precision was increased to 63.936%, recall was increased to 58.263%, mAP was increased to 62.45%, and there was also an increase in the F1 score.

In Ablation Experiment 8, all three modules are introduced simultaneously into the original network, resulting in a maximum improvement of approximately 1.4% in mAp50 compared to the original network. Furthermore, FLOPs were reduced by 0.2G, while simultaneously ensuring a balanced amount of precision and recall improvement, with approximately 0.5%, 1%, and 1.5% increases observed in these areas.

### 5.2. Comparison Experiment

**Figure 8:**
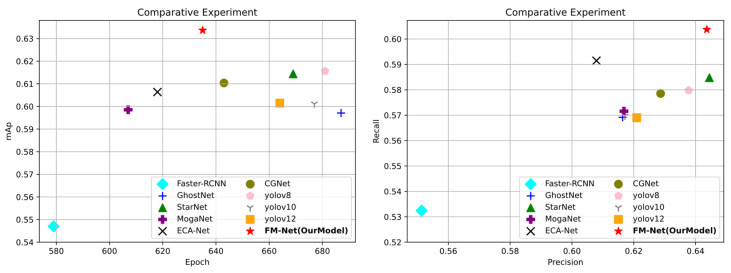
Scatterplot of comparative test results.

We trained the neural network model using the smoke/flame dataset constructed in this paper. As shown in Table 3 and Figure 8, under fair comparison conditions where no pre-trained models are used and all models are trained from the first round, the model FM-Net discussed in this paper achieves an mAp50 metric of 63.367%, which is state-of-the-art compared to other single-stage and two-stage detectors.

Faster R-CNN [28] is a typical and popular two-stage detection algorithm. Faster R-CNN, due to the specificity of its own network model, may destroy the continuity characteristics of texture-ambiguous targets such as smoke and flame in the process of regional feature extraction, and it is not sensitive enough to small target details. Its complex process also makes the model bulky, difficult to deploy in edge devices, and less flexible than lightweight single-stage models in adapting to dynamically changing flame scales. Even though the model in this paper has a slight lack of convergence speed, it significantly outperforms it in terms of accuracy, with 9% higher precision and 6% higher recall.

In addition, compared with the current state-of-the-art single-stage detection algorithms, the model in this paper achieves a better trade-off between convergence speed and detection accuracy. The performance of GhostNetv2 [29] and MogaNet [30] is relatively low, and the mAp50 index does not exceed the threshold of 0.6. Similarly, the low precision and recall indicate that their detection ability is limited at higher IoU thresholds. Even though ECA-Net, which has a slightly higher recall, has a corresponding change in F1 value, its detection ability is still in decline because its precision has just exceeded the threshold of 0.6. The precision and recall of StarNet [31] are more balanced, showing a better detection balance, perhaps due to the existence of an element-wise multiplication that realizes a high level of detection capability in the network architecture. Even so, its precision (0.64447) and recall (0.58478) are lower than our model.

Compared to the YOLO series, these models all have high convergence rounds, and as the versions iterate, while the convergence rounds continue to decrease, so do the mAp values (YOLOv8 F1 = 0.607389 => YOLOv10 F1 = 0.592473 => YOLOv12 F1 = 0.593851), and the best performer in the series is YOLOv8, with an F1 value of 0.607389, but still below the 2% modeled in this paper. YOLO has been able to stretch its speed advantage with the version change, but there are obvious shortcomings in smoke and flame detection. Its single detection mechanism and preset anchor frames have insufficient ability to capture small-sized, irregularly shaped initial smoke and flame targets, with a high leakage rate. At the same time, in order to pursue speed, its backbone network may be oversimplified or still need to be compressed on resource-constrained devices, resulting in limited feature extraction capability, as well as easy deterioration of false detection rate and localization accuracy in complex backgrounds and harsh environments, making it difficult to balance real-time and high-precision low-false-alarm requirements.

Analyzed from the perspective of floating-point number operations, the FLOPs of FM-Net are controlled at 9.2G, which is significantly lower than Faster R-CNN (134G) and ECA-Net (15.6G) and is comparable to the computational overhead of efficient models such as YOLOv8 and MogaNet. Under the premise of maintaining moderate computational overhead, the model achieves the best recall (0.604) and F1 score (0.623) in the whole field, fully reflecting the balanced advantages of computational efficiency and detection accuracy.

**Figure 9:**
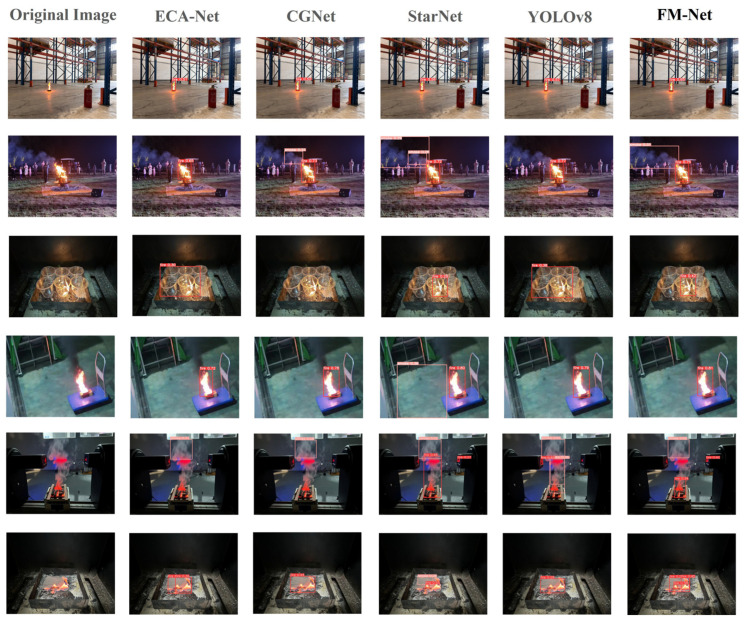
Smoke and flame prediction experiment.

In particular, this paper conducts smoke/flame prediction experiments on some of the above comparative models and the model designed in this paper after training, and the specific experimental results are shown in Figure 9.

The first line shows the experimental flame detection scene in the cargo warehouse; each detection algorithm labeled the flame with “fire”, but the confidence level was different—FM-Net’s flame detection confidence level was the highest, at 0.65. The second line shows a scene of the institute’s reunion bonfire party, and FM-Net detects the burning wood and gives a high confidence level of 0.75 correctly recognizes the overall outline of the burning white smoke. The third line shows a scene where six transparent cups are placed in an oil tray and a match is placed in one of the cups. The light from the fire penetrates through the transparent cups and causes interference by expanding the light source, but FM-Net still maintains a high confidence level of 0.42 and has the best labeled contours. The fourth row shows a scene of a burning oil pan on a laboratory trolley. All algorithms recognize the smoke and fire, but there are differences in the labeled positions, and FM-Net detects it with the highest confidence and without any false alarms. The fifth row shows the detection of smoke and fire in a specific experimental scenario; all algorithms recognize the smoke and fire but there is a difference in the confidence and location of the labeling. FM-Net again shows high confidence and performs the best in terms of outline labeling of the flame range. The sixth row shows a burning cotton material in an oil pan; all algorithms recognize the smoke and fire, but the labeled locations differ. FM-Net’s detection achieves the highest confidence and no false alarms.

### 5.3. Comparison on Fire-Flame-Dataset

**Figure 10:**
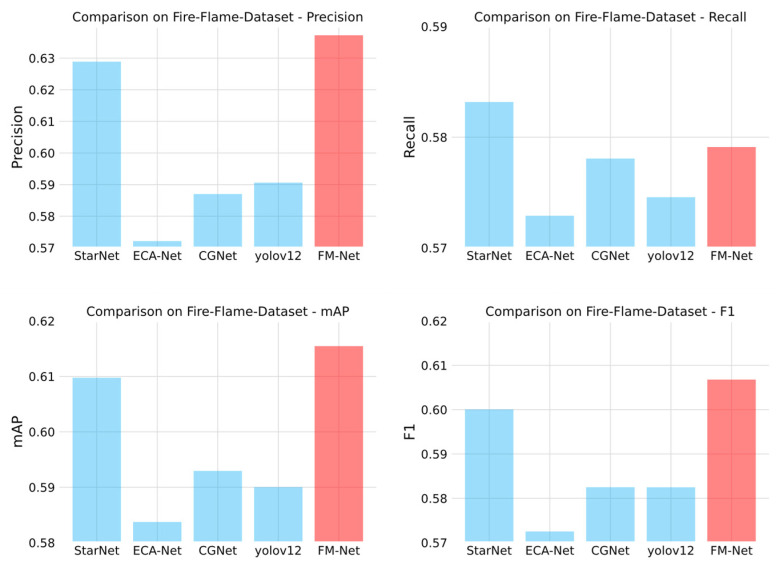
Bar chart of comparative test results on Fire-Flame-Dataset.

To further test the detection method designed in this paper, a publicly available dataset, the Fire-Flame-Dataset [32], is chosen to compare the results of the detection models. This is a dataset containing about 3000 images and three categories, where the labels include “fire”, ‘smoke’ and “neutral”, and each category has 1000 datapoints. In this paper, a total of 1800 images labeled as “fire” and “smoke” are used for model training and 200 images are used for model testing. In order to make a fair comparison, the experiment did not use a pre-trained model. FM-Net can achieve a 61.546mAp50 index, which represents a state-of-the-art performance compared with other general-purpose detection models. The results of the comparative experiments are shown in Table 4 and Figure 10.

As can be seen from the above table, the FM-Net model shows an overall leading performance advantage, with a precision rate of 0.63723, the highest value among all models; it also tops the list with an mAP50 metric of 0.61546 and an F1 score of 0.606781435. The StarNet model performs next best, obtaining a precision rate of 0.61888 and a 0.60976 mAP50, and its F1 score of 0.600499873 and recall of 0.58318 are at the second highest level. CGNet and the yolov12 model show similar performance characteristics: CGNet is slightly better than the yolov12 counterpart, with a precision rate of 0.58702, recall of 0.57807, and mAP50 of 0.59294 versus 0.59062, 0.57458 and 0.59002; however, the F1 scores of the two are extremely close to each other, 0.582510624 and 0.582489598, respectively. The ECA-Net model is at the bottom of the list of indicators, with a precision rate of 0.57213, a recall rate of 0.5729, an mAP50 of 0.58373, and an F1 score of 0.572514741, which are lower than the other models.

## 6. Conclusions

In this paper, a novel smoke and flame detection method called FM-Net is proposed, which innovatively integrates a Context Guided Convolutional Block, Poly Kernel Inception Block, and Manhattan Attention on the basis of the feature pyramid structure. The mechanism effectively enhances the model’s ability to perceive complex backgrounds, details and edge-blurred regions in fire images and improves the detection accuracy of smoke and flame targets. When dealing with smoke and flame, which are typical targets with low contrast, blurred edges and irregular morphology, the proposed method demonstrates stronger robustness and accuracy. The experimental results show that the proposed method outperforms existing mainstream detection models in several metrics, demonstrating superior overall performance. The fire detection algorithm proposed in this study provides new ideas for solving the key problems in practical applications and has important theoretical significance and application value.

Although FM-Net has made progress in the detection of fireworks in single visible light images and improved its perception capabilities in complex scenes, it still falls short when it comes to certain difficult targets. When faced with tiny transparent smoke, strong light suppressing flames, or environments that are highly similar to the background, the model has difficulty detecting targets or has low confidence, which stems from the inherent limitations of single visible light images, namely, their single information dimension. Tiny or low-concentration targets are easily assimilated into the background. In scenarios with strong light and color confusion, the spectral and visual features of targets and interference objects are similar, lacking distinguishing information. To enhance robustness in extreme scenarios, future efforts will focus on integrating more diverse information dimensions by introducing multispectral imaging and leveraging non-visible light features to enhance the discrimination of weak signals.

## Figures and Tables

**Table 1:** Information of the dataset.

Dataset	Training Set	Validation Set	Total
Single Flame Picture	3000	500	3500
Single Smoke Picture	3500	1125	4625
Smoke & Flame Picture	2500	1000	3500
Negative Picture	1000	0	1000
Total	10,000	2625	12,625

**Table 2:** Analysis table of ablation experiment results.

	Model A	Model B	Model C	Precision	Recall	mAP50	F1	FLOPs
1				0.63776	0.57978	0.6156	0.607389478	8.9G
2	**√**			0.64286	0.57555	0.61678	0.607345759	8.2G
3		**√**		0.63924	0.58277	0.61759	0.609700239	8.4G
4			**√**	0.6444	0.57054	0.61442	0.60522491	8.3G
5	**√**	**√**		0.63602	0.58262	0.62456	0.608150024	8.5G
6	**√**		**√**	0.64078	0.57307	0.61441	0.605036528	8.6G
7		**√**	**√**	0.63936	0.58041	0.6245	0.608460509	8.6G
8	**√**	**√**	**√**	0.64383	0.58996	0.62923	0.615718958	8.7G

Module A is the CGConv module, Module B is the PKIBlock module, and Module C is the MHTAttention module.

**Table 3:** Analysis table of comparative experimental results.

Name	Precision	Recall	mAP50	F1	FLOPs
Faster R-CNN	0.55135	0.53244	0.54702	0.54173	34G
GhostNet	0.61638	0.56916	0.59709	0.59183	5.8G
StarNet	0.64447	0.58478	0.61442	0.613176	8.9G
MogaNet	0.61683	0.57158	0.59852	0.593344	8.6G
ECA-Net	0.60793	0.59147	0.60637	0.599587	15.6
CGNet	0.62868	0.57854	0.61037	0.602569	8.6G
yolov8	0.63776	0.57978	0.6156	0.607389	8.9G
yolov10	0.61712	0.56972	0.60113	0.592473	7.8G
yolov12	0.62096	0.56901	0.60147	0.593851	7.3G
**FM-Net**	**0.64363**	**0.60374**	**0.63367**	**0.623047**	**9.2G**

**Table 4:** Analysis table of comparative experimental results on Fire-Flame-Dataset.

Name	Dataset	Precision	Recall	mAP50	F1
StarNet	Fire-Flame-Dataset	0.61888	**0.58318**	0.60976	0.600499873
ECA-Net	Fire-Flame-Dataset	0.57213	0.5729	0.58373	0.572514741
CGNet	Fire-Flame-Dataset	0.58702	0.57807	0.59294	0.582510624
yolov12	Fire-Flame-Dataset	0.59062	0.57458	0.59002	0.582489598
**FM-Net**	Fire-Flame-Dataset	**0.63723**	0.57911	**0.61546**	**0.606781435**

## Data Availability

The raw data supporting the conclusions of this article will be made available by the authors upon request.
